# Gene expression profiling of candidate virulence factors in the laminated root rot pathogen *Phellinus sulphurascens*

**DOI:** 10.1186/1471-2164-15-603

**Published:** 2014-07-17

**Authors:** Holly L Williams, Rona N Sturrock, Muhammad A Islam, Craig Hammett, Abul K M Ekramoddoullah, Isabel Leal

**Affiliations:** Natural Resources Canada, Canadian Forest Service, Pacific Forestry Centre, Victoria, V8Z 1M5 BC Canada

**Keywords:** Virulence, Pathogenicity, Laminated root rot (LRR), *Phellinus sulphurascens*, Douglas-fir

## Abstract

**Background:**

*Phellinus sulphurascens* is a fungal pathogen that causes laminar root rot in conifers, one of the most damaging root diseases in western North America. Despite its importance as a forest pathogen, this fungus is still poorly studied at the genomic level. An understanding of the molecular events involved in establishment of the disease should help to develop new methods for control of this disease.

**Results:**

We generated over 4600 expressed sequence tags from two cDNA libraries constructed using either mycelia grown on cellophane sheets and exposed to Douglas-fir roots or tissues from *P. sulphurascens*-infected Douglas-fir roots. A total of 890 unique genes were identified from the two libraries, and functional classification of 636 of these genes was possible using the Functional Catalogue (FunCat) annotation scheme. cDNAs were identified that encoded 79 potential virulence factors, including numerous genes implicated in virulence in a variety of phytopathogenic fungi. Many of these putative virulence factors were also among 82 genes identified as encoding putatively secreted proteins. The expression patterns of 86 selected fungal genes over 7 days of infection of Douglas-fir were examined using real-time PCR, and those significantly up-regulated included rhamnogalacturonan acetylesterase, 1,4-benzoquinone reductase, a cyclophilin, a glucoamylase, 3 hydrophobins, a lipase, a serine carboxypeptidase, a putative Ran-binding protein, and two unknown putatively secreted proteins called 1 J04 and 2 J12. Significantly down-regulated genes included a manganese-superoxide dismutase, two metalloproteases, and an unknown putatively secreted protein called *Ps*0058.

**Conclusions:**

This first collection of *Phellinus sulphurascens* EST sequences and its annotation provide an important resource for future research aimed at understanding key virulence factors of this forest pathogen. We examined the expression patterns of numerous fungal genes with potential roles in virulence, and found a collection of functionally diverse genes that are significantly up- or down-regulated during infection of Douglas-fir seedling roots by *P. sulphurascens*.

**Electronic supplementary material:**

The online version of this article (doi:10.1186/1471-2164-15-603) contains supplementary material, which is available to authorized users.

## Background

The basidiomycete *Phellinus sulphurascens* (*Ps*) is the causal agent of laminated root rot (LRR), one of the most damaging root diseases affecting conifers in northwestern America [1]. Douglas-fir (DF; *Pseudotsugata menziezii*) and grand fir (*Abies grandis*) are particularly susceptible to this disease [2]. Like most root pathogens, *Ps* can live as both a parasite and saprotroph. As a parasite, it initiates infection by colonizing the live bark and cambium of its host, then progressively moves inward to kill phloem and cambial tissues, spreading both proximally and distally from the point of infection. It then enters and initiates decay in the xylem, and finally advances into the heartwood and upward in the stem [3]. Wounding the roots facilitates but is not essential for parasitic colonization by the fungus [3]. As a saprotroph, *Ps* can remain viable in stumps, fallen stems, and roots for as long as 50 years [4]. New infections most commonly begin when new roots of susceptible hosts contact infested stumps or roots left in the soil from the previous stand [3]. It has been estimated that LRR decreases stand volume by 40 – 70% on infected sites [5].

As recommended in a recent report [6], a better understanding of the DF – *Ps* pathosystem should help to develop new methods for control. While significant progress has been made on genomic and proteomic aspects of the response by Douglas-fir to *Ps* infection [7–9], the fungus itself is poorly characterized at the molecular level. Aside from taxonomic and population studies comparing ITS regions [10], no gene sequences from this fungus have yet been reported. A proteomics study of DF response to *Ps* infection revealed one protein of fungal origin, 1,4-benzoquinone reductase, that was up-regulated during infection [9]. In the white rot fungus *Phanerochaete chrysosporium* this protein plays a critical role in vanillin metabolism, which is a key intermediate step in lignin biodegradation [11]. This finding suggests that lignin degradation may play an important role in the infection strategy of *Ps* [9].

A small but growing number of studies have examined genes expressed in conifer pathogens, including *Armillaria solidipes*, a white-rot root disease pathogen [12], *Grosmannia clavigera*, a mountain pine beetle-associated pathogen of lodgepole pine [13, 14], and *Heterobasidion annosum sensu lato*, a complex of closely related white-rot basidiomycetes that causes wood-decay in pine, spruce and fir trees [15–17], reviewed in [18]. In this study, we utilized expressed sequence tag (EST) sequencing to discover some of the fungal genes expressed during the *Ps*-DF interaction. To gain insight into the processes underlying *P. sulphurascens* pathogenesis and development, we constructed and analyzed two cDNA libraries: a pure fungal library of mycelia grown on cellophane sheets and exposed to Douglas-fir roots (*Ps* library), and a mixed library of DF roots infected with *Ps* (*Ps-*DF library). Our data represent the first available sequence information on genes expressed by this basidiomycetous fungus, and expand the public database of genes expressed in forest pathogens. The host plant genes expressed from the *Ps*-DF library have been reported separately [7, 8]. We identified several *Ps* genes putatively involved in pathogenicity based on homology to genes from other phytopathogens that have been shown to play a role in the infection process. Also, because many virulence factors are secreted, we identified which genes are likely to be part of the secretory pathway in *Ps.* Including two *Ps* genes sequenced separately from the cDNA libraries, 74 putative virulence factors and 46 other putatively secreted proteins were identified. We designed primers for 86 of these 120 genes to examine their expression patterns during the first 7 days of *Ps* infection of DF seedlings. Genes that showed significant up- or down-regulation in the initial screen were further assessed using 3 biological replicates. This work has identified 16 genes potentially important during *Ps* infection of DF. Future functional studies on the proteins encoded by these 16 genes will more definitively establish their role during the infection process.

## Results

### Titre of cDNA libraries

The primary *Ps*-DF library had a titre of 10^6^ cfu ml^−1^, and after amplification the titre was 3 × 10^9^ cfu ml^−1^. The titre of the *Ps* library was low, with only 118,000 clones in total. However, we checked a small sample of the clones, and estimated that 90% were recombinant, with insert sizes ranging from approximately 200 bp to 3 kb. Thirty randomly-selected clones were sequenced and we obtained 26 different high quality reads representing a variety of genes from several different functional categories.

### Contig assembly and analysis of *P. sulphurascens*ESTs

After assembly, the 1007 high quality sequences from the *Ps* library yielded 713 unisequences of which 579 were singletons and 134 were contigs containing 2 to 21 sequences. For the *Ps*-DF library, BLASTx analysis identified 443 sequences with significant hits to fungal genes. Assembly of these 443 sequences yielded 270 unisequences of which 205 were singletons and 65 were contigs containing 2 to 11 sequences. There were no non-overlapping sequences among the 5′ and 3′ fungal transcripts from the *Ps*-DF library. To assess the overlap between the *Ps* and *Ps*-DF libraries, we assembled all of the high quality fungal sequences from both libraries using Sequencher 4.8, and confirmed manually that 93 of the contigs contained sequences from both libraries. Thus 93 of 270 (34.4%) of the unisequences from the *Ps*-DF library were also present in the *Ps* library, and 93 of 713 (13%) of the unisequences from the *Ps* library were also present in the *Ps*-DF library. The 890 individual high quality fungal EST sequences from the two libraries were submitted to the dbEST database at NCBI: JK341455, JK341456, and JK316298 through JK317123 (unisequences from single-pass reads), and JO317757 through JO317818 (computationally assembled unisequences). A categorized list of all of the genes from the *Ps* and *Ps*-DF libraries are found in Additional files 1 and 2, respectively.

After trimming vector and poor quality sequence ends, the mean length of all the *Ps* reads was 652 bp, and the mean length of all the fungal *Ps*-DF Forward reads was 516 bp. Based on comparisons with homologous proteins in the public databases, 31% of the *Ps* library unisequences included complete open reading frames (ORFs), and 44% of the fungal unisequences from the *Ps*-DF library contained complete ORFs.

### Functional classification of *P. sulphurascens*genes expressed during induction by Douglas-fir roots

**Figure 1:**
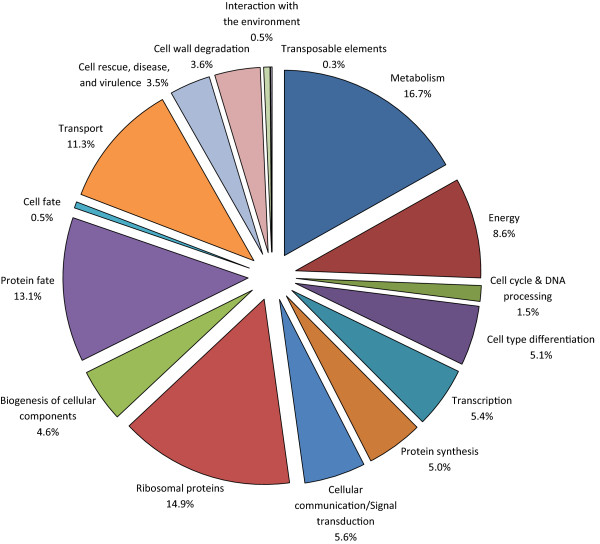
**Functional classification of 730 ESTs from the** ***P. sulphurascens*** **library with significant similarities to genes available in GenBank.** The putative genes were classified into functional categories following the Munich Information Center for Protein Sequences Functional Categories (MIPS FunCat) annotation scheme [56].

Functional annotation was possible for 72% of the 1007 ESTs (70% of the 713 unisequences) in the *Ps* library. In classifying the predicted proteins, we used the MIPS FunCat scheme with a few minor modifications. ‘Cell wall degradation’ , which in FunCat falls under the subcategory ‘Extracellular metabolism’ , was here designated as its own separate category; likewise ‘Ribosomal proteins’ , normally a subcategory of ‘Protein Synthesis’ , were also given their own category. The 1007 ESTs were divided among 18 broad functional categories, including an ‘Unclassified’ and a ‘No significant match’ category. Not including these last two groups, approximately 16.7% of the total annotated proteins were involved in metabolism, 13.1% in protein fate, 11.2% in transport, 8.6% in energy, 5.0% in protein synthesis, 3.6% in cell wall degradation, 3.5% in cell rescue, disease, and virulence, and 14.9% were identified as ribosomal proteins (Figure 1). Of the 25.8% of ESTs that could not be assigned to functional categories, 15.7% were similar to predicted proteins or to proteins for which a function has yet to be determined, and were designated as “unclassified”, and 10.1% did not exhibit significant similarity to any other sequences in the databases.

The most prevalent transcript in the *Ps* library, with 21 ESTs, encoded a predicted small (98 aa), secreted protein with no matches in the databases. The second and third most prevalent transcripts, with 15 and 14 ESTs, respectively, encoded two nearly identical hydrophobins. Four other hydrophobins (1 to 4 transcripts each) were also identified for a total of 38 hydrophobins. Other prevalent (≥5) transcripts included those with similarities to a putative galactose mutarotase-like protein (11 transcripts), translation elongation factor 1a (9), two unclassified proteins (7 transcripts of one, 6 of another that is putatively secreted), guanine nucleotide binding protein beta subunit (6 transcripts), manganese superoxide dismutase (5 transcripts), peptidyl-prolyl cis-trans isomerase (5 transcripts), an unclassified, putatively secreted protein (5 transcripts), and several ribosomal proteins (up to 7 transcripts each) (Table 1). A number of sequences matching endoglucanases and ubiquitin-conjugating enzyme E2 were identified, but most of these corresponded to families of related enzymes rather than identical copies of the same transcript. Eleven transcripts comprising five groups of metalloproteases were also identified.

**Table 1:** **Unique transcripts ranked by EST frequency in the** ***Ps*** **library** ^**a**^

***Ps***unisequence GenBank accession	EST frequency	Best match ^b^; accession no.	E-value
JK317046	21	No significant similarity	n/a
JK317028	15	Hydrophobin; ESK92236	2.E-29
JK317027	14	Hydrophobin; ESK92236	1.E-29
JO317760	11	Galactose mutarotase-like protein; EIW62403	0.0
JO317794	9	Translation elongation factor 1a; EMD41826	2.E-115
JK316963	7	Hypothetical protein; EJC98024	5e-47
JK317029	6	Hypothetical protein; EJD07754	7e-21
JO317801	6	Guanine nucleotide binding protein β subunit; EJD02729	0.0
JK317052	5	Manganese superoxide dismutase; EJD01959	4e-112
JK316977	5	Peptidyl-prolyl cis-trans isomerase; EJC99042	3e-64
JK317031	5	Hypothetical protein; EJD35586	2e-38

^a^ESTs matching to ribosomal proteins were excluded from this list.

^b^‘Best match’ determined using results of a BLASTx analysis against the nonredundant database.

### Functional classification of *P. sulphurascens*genes expressed during infection of Douglas-fir roots

**Figure 2:**
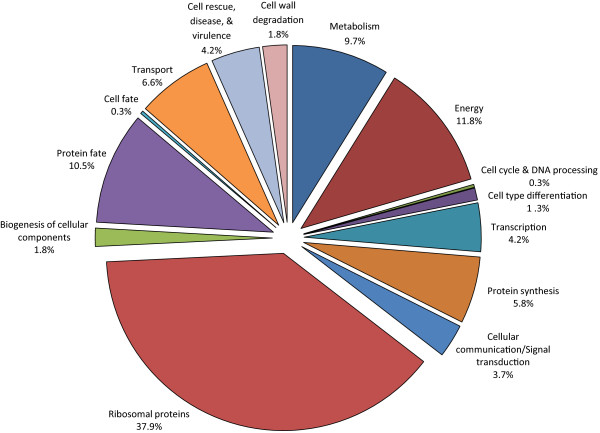
**Functional classification of 378 ESTs from the** ***P. sulphurascens-*** **Douglas-fir library with significant similarities to fungal genes available in GenBank.** The putative genes were classified into functional categories following the Munich Information Center for Protein Sequences Functional Categories (MIPS FunCat) annotation scheme [56].

Of the 446 ESTs from the *Ps*-DF library that shared significant similarity with fungal sequences in the public databases, functional annotation was possible for 85% (83% of the unisequences). We excluded all sequences with no significant similarities to known sequences (E-value > 10^−5^) in our analyses of this library, because it was uncertain whether they originated from *Ps* or DF. Of the 380 annotated ESTs, approximately 9.7% were involved in metabolism, 10.5% in protein fate, 6.6% in transport, 11.8% in energy, 5.8% in protein synthesis, 1.8% in cell wall degradation, 4.2% in cell rescue, disease, and virulence, and 37.9% were ribosomal proteins (Figure 2). Unclassified proteins accounted for 15% of the total fungal ESTs from this library.

The most prevalent fungal transcript in the *Ps*-DF library, with 11 copies, was a predicted protein that shared putative homology (E-value of 10^−14^) with DNA-binding helix-turn-helix proteins, which are transcriptional regulators. Other transcripts in relatively high abundance included a cytochrome-c oxidase subunit (5 transcripts), ubiquitin-conjugating enzyme E2 (5 transcripts), an unclassified protein (5 transcripts), a ubiquinol-cytochrome c reductase complex protein (4 transcripts), an unclassified protein (4 transcripts), and numerous ribosomal proteins (up to 9 transcripts each) (Table 2).

**Table 2:** **Unique transcripts ranked by EST frequency in the** ***Ps*** **-DF library** ^**a**^

***Ps***unisequence NCBI accession	EST frequency	Best match ^b^; accession no.	E-value
JK316958	11	Unclassified protein similar to DNA-binding helix-turn-helix protein; EUC56661	5.E-14
JK317101	5	Cytochrome-c oxidase; EJD07707	9.E-26
JK317036	5	Ubiquitin-conjugating enzyme E2; XP_001832449	2.E-101
JK316397	5	Hypothetical protein; EIW51282	4.E-49
JK316305	4	Ubiquinol-cytochrome c reductase complex; EJD05913	3.E-75
JK316404	4	Hypothetical protein; EJD05568	5.E-15

^a^ESTs matching to ribosomal proteins were excluded from this list.

^b^‘Best match’ determined using results of a BLASTx analysis against the nonredundant database.

### ESTs predicted to be involved in virulence

We identified genes for which there is evidence for a role in the infection process in other pathogens, based on a search of the relevant literature. Seventy-nine *Ps* genes identified as candidate virulence factors are listed in Additional file 3. We considered all proteins classified in the functional categories “Cell wall degradation” and “Cell rescue, disease and virulence” to be potential virulence factors. Because proteins directly involved in virulence tend to be small and secreted, we identified all open reading frames with a N-terminal signal peptide that make them eligible for secretion from the fungus. Eighty-two (9.2%) of the 890 unisequences from the two libraries were predicted to encode secreted proteins (Additional file 4).

Two additional *Ps* sequences encoding for putatively secreted proteins identified apart from the current study (H.L.Williams, unpublished data) were added to the list of potential virulence factors: a 1,4 benzoquinone reductase, originally identified as a protein up-regulated during infection of DF [9] (partial gene sequence in Additional file 5), and a cyclophilin, which was identified as a prevalent protein in *Ps* mycelia grown on malt extract [GenBank: JX848541].

In most cases, the putative function given in Tables 1 and 2 and Additional files 1 through 4 is that of the protein with the lowest E-value (highest similarity) in the BLASTx search. Where the highest similarity was to a hypothetical protein of unknown function, the putative function of the top match having an assigned name (if available) and its E-value are given. For redundant ESTs, the clone with the lowest E-value is reported.

### Expression of putative virulence genes

**Figure 3:**
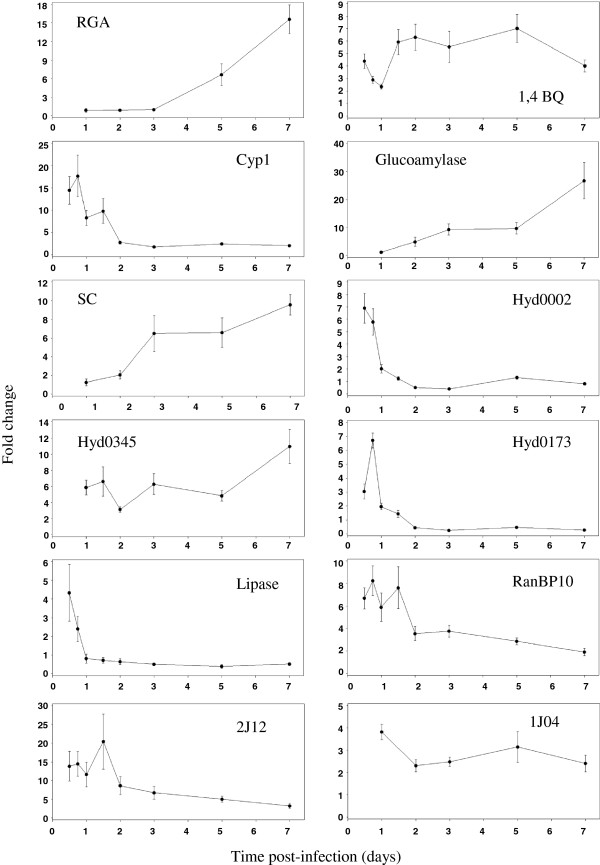
**Gene expression profiles of 12** ***P. sulphurascens*** **genes up-regulated in infected Douglas-fir seedling roots.** By qRT-PCR with ubiquitin conjugating enzyme E2 (*Ps uceE2*) as an internal control gene, relative quantities of the 12 genes were measured in vegetative mycelia and infected DF seedling roots collected at eight time points from 12 hours to 7 days post-inoculation. This experiment was performed with three biological replicates with triplicate technical replicates. Error bars represent standard deviations. RGA = rhamnogalacturonan acetylesterase, 1,4BQ = 1,4 benzoquinone reductase, SC = serine carboxylesterase, Cyp = cyclophilin, Hyd = hydrophobin, RanBP10 = Ran-binding protein 10.

**Figure 4:**
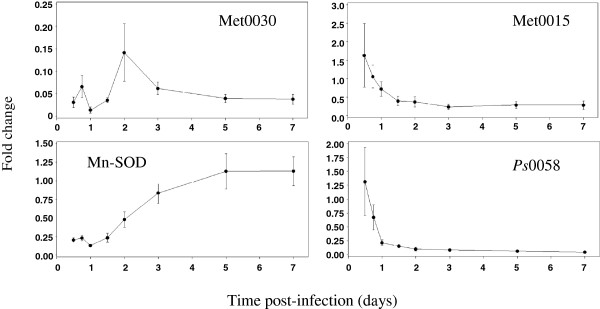
**Gene expression profiles of four** ***P. sulphurascens*** **genes down-regulated in infected Douglas-fir seedling roots.** By qRT-PCR with ubiquitin conjugating enzyme E2 (*Ps uceE2*) as an internal control gene, relative quantities of the four genes were measured in vegetative mycelia and infected DF seedling roots collected at eight time points from 12 hours to 7 days post-inoculation. This experiment was performed with three biological replicates with triplicate technical replicates. Error bars represent standard deviations. Met = metalloprotease, Mn-SOD = manganese superoxide dismutase.

**Figure 5:**
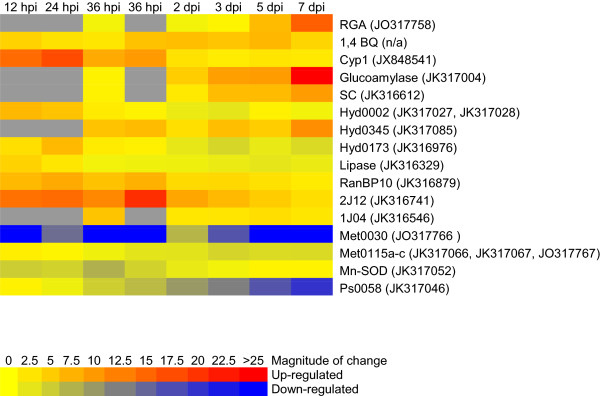
**Heat map showing expression levels of 16** ***P. sulphurascens*** **genes during infection of Douglas-fir seedling roots.** Expression levels were determined by qRT-PCR and are relative to those in vegetative mycelia. Full gene names and numerical values for the fold changes and P-values are shown in Table 3.

The outcome of our initial screen for infection-responsive expression patterns in 86 candidate virulence genes resulted in the identification of 16 candidate fungal genes that may be important in the virulence of *Ps*. These 16 genes were significantly up- or down- regulated during infection of DF seedling roots (Table 3 and Figures 3, 4 and 5). The earliest up-regulated genes at 12 hpi were cyclophilin 1 (14.4×, p = 0.0005), lipase (4.3×, p = 0.0430), 1,4 benzoquinone reductase (4.4×, p < 0.0001), a putative Ran-binding protein (6.7×, p < 0.0001) and an unknown gene called 2 J12 (13.8×, p = 0.0056)*.* While the increase in lipase levels was short-lived, cyclophilin 1, the putative Ran-binding protein, and 2 J12 sustained high levels of up-regulation during the initial 36 hpi, and 1,4 benzoquinone reductase maintained fairly steady levels throughout the 7 day infection period. The greatest increase in transcription levels was for the glucoamylase gene, which gradually increased from 4.9× at 2 dpi (p = 0.0842) to 26.8× at 7 dpi (p = 0.0003). Similarly, serine carboxypeptidase (SC) showed a gradual up-regulation beginning around 3 dpi and reaching 9.6× at 7 dpi (p = 0.0021). Rhamnogalacturonan acetylesterase (RGA) was also highly up-regulated (15.6×, p <0.0001) at the final 7 dpi time point. Three hydrophobins showed varying patterns of up-regulation; Hyd0345 expression levels were too low for detection at 12 hpi and 24 hpi and then sustained at approximately 6-fold up-regulation levels for the rest of the time course (p < 0.017), while Hyd0002 and Hyd0173 were briefly up-regulated to 6.9× (p = 0.0002) and 6.7× (p < 0.0001), respectively, during the first 24 hpi. A gene called 1 JO4 with low homology to a MAPK-interacting protein maintained somewhat stable albeit low levels of up-regulation throughout the infection time course (Table 3; Figures 3 and 5).

**Table 3:** **Gene expression (fold changes) of** ***P. sulphurascens*** **genes significantly up- or down-regulated during infection of DF**

	Fold change over time (P) ^a^							
Gene	12 hpi	24 hpi	36 hpi	36 hpi	2 dpi	3 dpi	5 dpi	7 dpi
Rhamnogalacturonan acetylesterase	n/a^b^	n/a	0.89 (0.0047)	n/a	0.91 (0.2438)	0.99 (0.9645)	6.63 (0.0284)	15.59 (<0.0001)
1,4 benzoquinone reductase	4.40 (<0.0001)	2.90 (<0.0001)	2.36 (0.0003)	5.93 (<0.0001)	6.31 (<0.0001)	5.54 (0.0010)	7.02 (0.0011)	4.02 (0.0013)
Cyclophilin 1	14.44 (0.0005)	17.63 (0.0021)	8.29 (0.0026)	9.79 (0.0119)	2.79 (0.0004)	1.80 (0.0026)	2.47 (0.0029)	2.08 (0.0118)
Glucoamylase	n/a	n/a	1.25 (0.4004)	n/a	4.86 (0.0842)	9.33 (0.0015)	9.70 (0.0220)	26.75 (0.0003)
Serine carboxyesterase	n/a	n/a	1.26 (0.4590)	n/a	2.06 (0.1812)	6.49 (0.0356)	6.58 (0.0336)	9.57 (0.0021)
Hydrophobin 0002	6.90 (0.0002)	5.79 (0.0011)	2.02 (0.0335)	1.24 (0.6378)	0.52 (0.0141)	0.40 (0.0189)	1.31 (0.5690)	0.82 (0.3056)
Hydrophobin 0345	n/a	n/a	5.87 (0.0117)	6.62 (0.0058)	3.13 (0.0065)	6.28 (0.0034)	4.82 (0.0163)	10.98 (0.0125)
Hydrophobin 0173	3.04 (0.0015)	6.72 (<0.0001)	1.96 (0.0339)	1.45 (0.4555)	0.44 (0.0003)	0.25 (<0.0001)	0.47 (0.0004)	0.28 (0.0002)
Lipase	4.33 (0.0430)	2.40 (0.0921)	0.81 (0.3147)	0.72 (0.3210)	0.65 (0.7940)	0.51 (0.1006)	0.40 (0.0055)	0.52 (0.0493)
Ran-binding protein 10	6.70 (<0.0001)	8.25 (<0.0001)	5.90 (0.0011)	7.63 (0.0016)	3.54 (0.0001)	3.77 (<0.0001)	2.86 (<0.0001)	1.91 (0.0591)
2 J12	13.83 (0.0056)	14.48 (0.0014)	11.63 (0.0009)	20.46 (0.0057)	8.62 (0.0006)	6.72 (0.0003)	4.99 (0.0001)	3.16 (0.0008)
1 J04	n/a	n/a	5.73 (0.0014)	n/a	2.30 (0.1030)	2.47 (0.0451)	3.14 (0.2431)	2.40 (0.1504)
Metalloprotease 0030	0.03 (<0.0001)	0.07 (<0.0001)	0.01 (<0.0001)	0.03 (<0.0001)	0.14 (0.0005)	0.06 (0.0003)	0.04 (0.0007)	0.038 (0.0002)
Metalloproteases 0115a-c	1.63 (0.3198)	1.06 (0.3916)	0.72 (0.9373)	0.39 (0.0911)	0.37 (0.5796)	0.24 (0.0787)	0.29 (0.0072)	0.28 (0.0002)
Mn-superoxide dismutase	0.20 (<0.0001)	0.23 (<0.0001)	0.13 (<0.0001)	0.23 (<0.0001)	0.48 (0.0001)	0.84 (0.0916)	1.13 (0.9779)	1.13 (0.5432)
*Ps*0058	1.32 (0.6118)	0.67 (0.7184)	0.21 (0.0150)	0.15 (0.0201)	0.10 (0.0222)	0.08 (0.0254)	0.06 (0.0066)	0.05 (0.0047)

^a^Fold-change values were obtained from quantitative reverse-transcriptase polymerase chain reaction expression data (crossing threshold values) analyzed by SAS PROC GLM repeated measures analysis of variance. P values obtained from differences between control mycelia and *P. sulphurascens*-infected DF.

^b^ *n/a* = Not applicable; qRT-PCR amplification not reliable because fungal transcript levels were too low at some of the earlier points in the infection time course.

A metalloprotease called Met0030 was immediately and intensely repressed approximately 25-fold throughout the time course, while 3 highly homologous metalloproteases (Met0115 a-c), were slightly repressed during the second half of the infection trial. A manganese-superoxide dismutase (Mn-SOD) was down-regulated throughout the first 2 dpi, and an unknown gene called *Ps*0058 was increasingly down-regulated from 24 hpi to 7 dpi (Table 3; Figures 4 and 5).

## Discussion

A number of studies have assessed defence-related gene expression in coniferous trees in response to root and butt rot fungi eg. [7, 8, 19]. Less is known about the fungal component of these pathosystems, although recent studies have begun to shed light on molecular strategies utilized by the pathogens [12–19]. The current study represents the first analysis of genes expressed by the causal agent of laminated root rot, *Phellinus sulphurascens*. The aim of this study was to enhance our understanding of LRR and improve management of the disease in affected forests.

### Genes up-regulated during the initial phase of plant infection

In hemibiotrophic and biotrophic pathogens, genes that regulate the development of infection structures and prevent elicitation of host defenses are among the first to be induced, whereas in nectrotrophic fungi, toxins and cell wall degrading enzymes are often among the first to be up-regulated [20]. In this study we were most interested in the genes induced during initial penetration of *Ps* into the roots of living trees and the early parasitic phase, with the goal of identifying candidate virulence factors that may be manipulated to halt or attenuate the symptoms of laminated root rot.

Our expression analyses indicated significant up-regulation for three hydrophobins in the initial stages of parasitic infection [GenBank: JK317027 & JK317028, JK316976] with one having sustained up-regulation throughout the seven day infection time course [GenBank: JK317085]. Hydrophobins are small, secreted fungal proteins found on the outer surfaces of cell walls of hyphae and conidia. These proteins assemble at hydrophobic/hydrophilic interfaces and thus may be involved in adhesion of fungi to host structures [21]. It has also been suggested that hydrophobins might act as ‘stealth’ factors, protecting an invading fungus from detection and rejection by its host plant [22]. In the rice-blast fungus *M. grisea*, two hydrophobin genes play essential roles in surface hydrophobicity and infection-related fungal development, and are required for virulence of *M. grisea* [23, 24]. In *H. annosum* s.s, a conifer pathogen with a similar life history to *Ps*, 16 hydrophobin-encoding genes with different regulatory patterns were observed. Several were highly up-regulated during saprotrophic growth on bark, sapwood and/or heartwood of Scots pine, suggesting that this pathogen employs different hydrophobins during growth on diverse wood components [25]. A separate study found that two *H. annosum* hydrophobin genes are up-regulated in aerial hyphae but are down-regulated during early infection of pine seedling roots [26]. It is clear that some hydrophobins play a crucial role in fungal pathogenesis, while others are needed for other biological processes.

Cyclophilins are peptidyl-prolyl cis–trans isomerases that are highly conserved throughout eukaryotes and have been implicated in a wide variety of cellular processes, including the response to environmental stresses and the control of transcriptional repression [27]. In *M. grisea*, disruption of cyclophilin highly expressed during plant infection resulted in a mutant strain impaired in appressorium turgor generation, penetration peg formation, and ability to infect rice seedlings [28]. In the chestnut blight fungus *Cryphonectria parasitica*, disruption of a cyclophilin gene revealed that it is required for virulence [29]. We identified a cyclophilin homolog (*cyp2*) in our *Ps* library, and previous protein work in our laboratory identified a paralogous cyclophilin sharing 71% coding sequence identity with the one from the *Ps* library (H.L.Williams, unpublished data). This cyclophilin (cyp1) [GenBank: JX848541] showed significant up-regulation during the earliest stages of the infection process, indicating that it may contribute to the virulence of *Ps.*

One of two *Ps* lipase genes [GenBank: JK316329] was significantly up-regulated at the beginning of the infection process, and may be involved in using DF triglycerides as a carbon source. In *Fusarium graminearum*, targeted gene disruption demonstrated that a secreted lipase encoded by *FLP1* is a major virulence factor of this fungus on wheat and maize [30], and in two obligate biotrophic rust fungi, secreted lipase transcripts were strikingly enriched (≥10-fold) *in planta* [31].

A gene sharing significant homology with Ran-binding protein 10 (RanBP10) [GenBank: JK316879] from *Rhizoctonia solani* was immediately up-regulated and sustained at high levels for the first 36 hpi. RanBP10 acts as a guanine nucleotide exchange factor for RAN GTPase, a master regulatory switch that controls shuttling of proteins between nuclear and cytoplasm compartments. Although the function of RanBP10 in fungi is not well studied, up-regulation of this gene in *Ps* during the initial stages of infection of DF suggests that it may play a key role in mobilizing intracellular signalling pathways required for the fungus to invade host tissues.

Given that secreted effectors tend to be small and poorly conserved between pathogens [31, 32], the characterization of small unknown secreted proteins may be fruitful in discovering novel virulence factors. We identified 20 functionally unclassified proteins with predicted signal peptides, three of which have no known homolog in the sequence databases. An unknown predicted 224 aa protein called 2 J12 [GenBank: JK316741] was highly up-regulated early in the infection of DF seedlings, and a predicted 220 aa protein called 1 JO4 with slight homology to a MAPK-interacting protein [GenBank: JK316546] maintained a low level of up-regulation throughout the infection time course. We aim to further characterize these unknown proteins in order to determine what role they play in the virulence of *Ps*. In particular, 2 J12 was cumulatively the most highly up-regulated of all the candidate genes over the seven day infection period, and was immediately up-regulated at high levels that were sustained for the first 36 hours of the infection time course. The 2 J12 protein is highly homologous to several unclassified proteins from *Fomitiporia mediterranea*, a wood decaying fungus belonging to the same order as *Phellinus*.

### Genes induced and expressed during the parasitic growth phase

White-rot fungi are unique among most microorganisms in their capacity to depolymerize and metabolize lignin. *Phellinus pini* (current name: *Porodaedalea pini*) and *H. annosum* are examples of fungi that remove the lignin at a faster rate than cellulose or the hemicelluloses in early decay stages [33]. This may also be the case for *P. sulphurascens*, given its close relationship and similar life history to both of these species. Our previous work on proteins regulated in DF in response to *Ps* [9] identified an up-regulated fungal protein that shared homology with a fungal 1,4-benzoquinone reductase, an enzyme involved in degradation of lignin in its host [11]. Expression analysis in our pathosystem confirmed that this gene was significantly up-regulated throughout the 7 day infection time course. We did not identify any lignin-degrading enzymes in the cDNA libraries, even though lignin is an important component of DF cell walls, possibly because the DF seedling roots were too young for substantial lignification to have occurred. In nature, *Ps* causes severe damage on mature, suberized roots, and some differences are expected between infection events in sterile three-week old seedlings and in larger roots in the forest. However, the fact that our study verified immediate up-regulation of a fungal enzyme involved in lignin degradation in young, scantly lignified seedlings suggests that lignin degradation could be important during the parasitic phase of *Ps* growth in DF. In *H. annosum* P strains, which are more aggressive pathogens and wood decayers than S strains, the oxidizing ligninolytic enzyme laccase is produced 5 to 6 times more than in S strains [34].

In addition to 1,4BQ, several other genes that were immediately induced remained up-regulated well after initial penetration of the host (i.e. after 2 dpi), including one hydrophobin (Hyd0345), the putative Ran-binding protein 10, cyclophilin 1, and the unknown secreted proteins 2 J12 and 1 J04. In addition to possible roles in recognition, adhesion or penetration of the host, the proteins encoded by these genes may be important in sustaining infection during the parasitic phase of *Ps* on DF roots.

### Genes induced during the later stage of parasitic growth

Our EST libraries revealed an array of glycoside hydrolase enzymes involved in extracellular plant cell wall degradation (CWDE), including the pectin-degrading enzymes exopolygalacturonase, pectinesterase, and rhamnogalacturonan acetylesterase, the hemicellulose-degrading enzyme endoxylanase, and the cellulose-degrading enzymes β-glucosidase and endoglucanase. We also identified four members of the recently characterized AA9 copper dependent LPMOs (formerly GH61), which support the activity of glycoside hydrolase enzymes through an oxidative degradation of cellulose [35]. However, our initial gene expression screen indicated induction of only one of enzyme involved in cell wall degradation: rhamnogalacturonan acetylesterase (RGA) [GenBank: JO317758], which was highly up-regulated at the end of the seven-day time course. Rhamnogalacturonan is a pectin polymer that constitutes a major portion of conifer cell walls [36], and RGAs are enzymes which in *Aspergillus aculeatus* have been shown to act in synergy with rhamnogalacturonases to degrade plant cell wall rhamnogalacturonan [37]. CWDE have been demonstrated to be virulence factors in several plant pathogenic fungi reviewed in [38]; however partially degraded polymers released by CWDE can act as elicitors of plant defense [39]. Biotrophic and hemibiotrophic fungi may need to carefully time the release of CWDE, possibly delaying their up-regulation until a sufficient mass of hyphae have penetrated the host tissues using other routes. This may be the case in *Ps*, given that the one induced enzyme was only up-regulated at 7 dpi. In the young seedlings used in our study, 7 dpi was considered to be part of the later parasitic stage, because at this point some of the DF roots were becoming weak and easily torn. The large increase in RGA expression suggests that degradation of plant cell wall pectin may be an important aspect of host colonization, and it is likely that a number of other CWDEs, including those not identified in this study, are also involved in degrading DF cell walls during the later parasitic and saprotrophic phases.

Much research on parasitism has focused on understanding pathogenicity and virulence factors, while the strategies used by parasites to acquire nutrients and adapt to changing nutritional environments are less well studied. A *Ps* glucoamylase [GenBank: JK317004] showed increasing up-regulation levels throughout the seven day infection period, and was the most highly up-regulated *Ps* gene at 7 dpi, indicating that this enzyme may be associated with the virulence of this fungus in DF. A glucoamylase secreted by *Sclerotinia sclerotiorum* was proposed to be important in providing energy to this phytopathogen via plant starch degradation during infection [40].

Extracellular proteases can play an active role in pathogenesis, and we observed a putatively secreted serine carboxypeptidase (SC) [GenBank: JK316612] was increasingly up-regulated from 3 dpi to the end of the infection time course. SCs have diverse biological functions, including supplying amino acids and energy to an entomopathogenic fungus during starvation and pathogenesis [41]. In the rice blast fungus *Magnaporthe grisea*, a serine carboxypeptidase was up-regulated during inductive appressorium formation [42]. A recent genome-wide association study of the necrotrophic forest pathogen *Heterobasidion annosum* sensu stricto (s.s.) yielded an SNP marker associated with virulence that was positioned beside a serine carboxypeptidase gene, suggesting a possible role for this enzyme in virulence [43].

### *Ps*genes down-regulated during infection of DF

One of the earliest plant responses to pathogen attack is a rapid, transient production of a large amount of reactive oxygen species (ROS), termed an ‘oxidative burst’ [44]. This represents a potentially toxic insult that may serve not only to protect the plant from the invading pathogen, but also as a signal to activate further plant defense reactions [45]. We identified several *Ps* ESTs corresponding to putative proteins that respond to oxidative stress, including a manganese-superoxide dismutase (Mn-SOD) [GenBank: JK317052]. Superoxide dismutases are believed to be responsible for directly inactivating ROS, although gene inactivation of an SOD gene in *Botrytis cinerea* indicated that this H_2_O_2_-generating enzyme is a virulence factor [46], contributing to the accumulation of phytotoxic H_2_O_2_ levels. However, our expression analysis of the *Ps* Mn-SOD gene showed that it was significantly down-regulated immediately upon infection of DF roots, followed by a gradual increase back to baseline (negative control) levels from two days to the end of the seven day infection period. This contrasts with findings in a similar pathosystem, where after 72 hours in contact with pine seedlings, Mn-SOD expression in *H. annosum* was almost double compared to that in controls [47]. However, when *H. parviporum* was inoculated on Norway spruce tissue cultures, the SOD expression profile was almost identical to ours [19]. The authors hypothesized that the SOD functions in protecting the fungal cell against superoxide produced as a by-product of oxidative metabolism, and that SOD activity should correlate with the nutritional status of the fungus, which is expected to be high during growth on the medium used for the controls and during growth on dead host cells in the later stages of host colonization. This would also explain the immediate repression and then gradual increase in Mn-SOD transcript levels over our infection time course. Mn-SOD therefore may not be a virulence factor in *Ps*, but may be important during nutrient acquisition in the later parasitic phase of its growth in DF.

We identified several other proteins involved in stress response and detoxification of xenobiotic substances, including thioredoxins, glutathione transferases, heat shock proteins, and cytochrome P450s, but due to sample limitations they have not yet been assessed for responsiveness during infection of DF using qRT-PCR. Our future work will examine whether antioxidant and detoxification gene expression is modulated when *Ps* is introduced to DF seedlings.

A number of transcripts comprising five groups of zinc-dependent metalloproteases were identified in the *Ps* library. Metalloproteases from two diverse plant pathogens are associated with virulence [48, 49], and given their prevalence in the *Ps* library and putative secretory signals, we considered these enzymes to be prime candidates as virulence factors. It was surprising that a *Ps* metalloprotease was greatly down-regulated throughout the entire seven day time course [GenBank: JO317766], and a second set of three nearly identical metalloproteases [GenBank: JK317066, JK317067, JO317767] were significantly down-regulated in the latter half of the infection trial. Although their down-regulation during the infection time course indicates these enzymes lack a key role in the pathogenic process, the answer to the question of why they are repressed during pathogenesis could be illuminating to our understanding of the pathogenic process. Metalloproteases are a broad and highly diverse group of enzymes, and it is difficult to hypothesize why they might be down-regulated in *Ps* during infection of DF. We found no evidence of similar repression of these enzymes in the literature.

The most prevalent transcript identified from the *Ps* library, a putatively secreted protein called *Ps*0058 [GenBank: JK317046], was increasingly and substantially down-regulated throughout the infection time course. This transcript shared no homology with any sequences in the NCBI database.

### Future work

Future studies aimed at the functional characterization of genes reported here via expression vector and/or targeted deletion experiments will help to elucidate whether the corresponding proteins are important in the virulence of *Ps*. We aim to identify genes that could prove to be effective targets for control of this pathogen. Additionally, these candidate genes may contribute to our understanding of the defense mechanisms and gene response of DF to *Ps*. This information may be useful for the development of molecular markers for marker-assisted breeding and screening of DF that are tolerant to *Ps*.

## Conclusions

In this study we report the first set of annotated *P. sulphurascens* EST sequences, a total of 890 unique sequences from two cDNA libraries. The putative identity of the majority these transcripts revealed many potential candidate genes that may be essential in the *Ps*/DF pathogenicity process. Analysis of the expression patterns of 86 of these genes during infection using real-time PCR revealed 16 fungal genes that are significantly up- or down-regulated during infection of DF. In conclusion, this work provides an important inventory of *P. sulphurascens* genes for exploring cellular events related to host-pathogen interactions.

## Methods

### Plant and fungal isolates and inoculation technique

Seed from coastal DF family 60686 were obtained from the Tree Seed Centre of the British Columbia Ministry of Forests and Range, located in Surrey, BC, Canada and stored in plastic bags at -20°C. To germinate the seeds, they were first soaked in ddH_2_O for 24 h, stratified at 4°C for 21 days, surface sterilized in 35% aqueous H_2_O_2_ (vol/vol) for 15 min with vigorous agitation, and rinsed four times with sterile H_2_O. Sterilized seeds were placed in petri plates (~20 seeds per plate) containing 10 g/l water agar (Sigma-Aldrich Canada Ltd), the plates were sealed with paraffin film, and the seeds were allowed to germinate in darkness at 24 ± 1C for 14 to 20 days, until most of the roots were at least ~ 4 cm long and the needles were no longer encased in the seed coat.

*P. sulphurascens* isolate PFC-581 was originally collected from the infected stem of a mature DF tree growing near Cowichan Lake on Vancouver Island, British Columbia (BC) in 1993. The fungus was isolated from the DF stem onto petri plates containing 15% malt extract agar (Sigma-Aldrich Canada Ltd, Oakville, ON, Canada) and then stored as mycelial plugs in cryovials filled with sterile double-distilled water at 3°C, at the Pacific Forestry Centre in Victoria, BC. Isolate PFC-583 was originally collected from a DF tree at Shawnigan Lake BC in 1993, and was cultured and stored in the same manner as PFC-581.

In preparation for creating a purely fungal cDNA library (*Ps* library), PFC-581 mycelial plugs were transferred to 15 g/l malt extract agar petri plates overlaid with a sterile cellophane sheet (Bio-Rad Laboratories, Mississauga, ON) and allowed to grow in darkness at room temperature for 3 weeks. The cellophane sheets (covered in mycelia) were then transferred to 15 g/l water agar plates and incubated at room temperature for 2 days. A portion of cellophane and the water agar beneath were cut and discarded (approx. 1/3 of each plate), and 12 DF seedlings were placed with their roots resting on the mycelia and their foliage resting in the empty portion of the plate. A second semi-circle of water agar with cellophane and mycelia was placed upside down over the roots of all the seedlings to form a sandwich. Petri plates were sealed with paraffin film, the portion containing the roots was covered with aluminum foil, and the plates were incubated in a growth chamber at 20°C with a photoperiod of 16 h (50 to 60 μmol m^−2^ s^−1^). We used cellophane sheets because they allowed sufficient quantities of fungal mycelia to be easily collected without inadvertent agar contamination. Additionally, cellophane sheets are composed of regenerated cellulose, and therefore may mimic some of the characteristics of plant cell walls.

We also created a *P. sulphurascens*-infected Douglas-fir cDNA library (*Ps*-DF library), the details of which have been described [8]. The inoculation method was essentially the same as for the *Ps* library, except the mycelial plugs were laid directly onto petri plates containing 1% water agar with no cellophane. The plates were incubated in darkness at room temperature for 20 days before seedlings were added.

### RNA isolation and cDNA library construction

Previous studies in our laboratory with scanning electron microscopy showed that using our infection method, *Ps* hyphae had penetrated DF seedling epidermal tissues by 2 dpi [50]. Necrosis of DF tissues was visible at 5 dpi, and light microscopy of prepared slides of *Ps*-infected roots stained with toluidine blue showed extensive mycelial colonization and hyphal penetration in the root cortex at 7 dpi [51]. These results suggested that optimal detection of pathogenicity-related gene expression would be between 2 and 7 dpi.

For the induced, purely fungal library (*Ps* library), infection sandwiches were dismantled, seedlings were discarded, and mycelia was scraped from both the upper and lower cellophane sheets using an RNase-free spatula. The mycelia was immediately frozen in liquid nitrogen, ground to a fine powder using a mortar and pestle with liquid nitrogen, and total RNA was extracted using the RNeasy Plant mini kit (Qiagen, Valencia, CA). Separate extractions were done for 2, 4 and 6 dpi induced mycelia, and the RNA was checked for quality and quantity using a Nanodrop 1000 spectrophotometer (Thermo Scientific, Wilmington, DE) and 1.2% agarose gel. To eliminate most of the ribosomal RNA, mRNA was isolated from total RNA by two successive mRNA extraction cycles with Dynabeads (Invitrogen Canada Inc, Burlington, ON). Again, the concentration and purity of the mRNA was checked using the Nanodrop spectrophotometer, and its integrity was verified on a 1.2% agarose gel.

For the *Ps* -infected DF library, RNA was extracted from infected DF roots at 3, 5, and 7 dpi using an RNA extraction protocol designed for use with recalcitrant plant (eg. conifer) tissues [8].

Both the purely fungal library and *Ps*-infected DF library were constructed using the Creator SMART cDNA library construction kit (Clontech Laboratories Inc, Mountain View, CA). For the fungal library, equal amounts of the 2, 4, and 6 dpi mRNA were pooled for a total of 1 μg mRNA. For the infected DF library, the 3, 5, and 7 dpi total RNA were pooled for a total of ~ 100 ng total RNA. These RNA samples were used for cDNA synthesis following the instructions of the manufacturer. Recombinant pDNR-LIB plasmids containing cloned cDNAs were transformed into *Escherichia coli* (ElectroMAX DH10B electrocompetent cells; Invitrogen, Carlsbad, CA) following the manufacturer’s instructions. To evaluate the quality of the cDNA libraries, 50 individual cDNA clones were randomly picked from each library and screened using M13 primers to determine the percentage of recombinant clones. We also initially sequenced 30 clones from the *Ps* library and 100 clones from the *Ps*-DF library, to ensure that the libraries contained a wide variety of gene sequences. The library titres were calculated following Clontech’s instruction manual. The mixed *Ps*-DF library was amplified following Clontech’s instruction manual.

### DNA sequencing, editing, and bioinformatics

We randomly selected 1400 *E. coli* clones from our induced *Ps* library and grew overnight cultures in 3 ml LB + 30 μg/ml chloramphenicol media at 37°C at 200 rpm. One μl of each overnight culture was used as PCR template with the M13 primer set. Clones with no insert or very short inserts (< ~100 bp) were discarded, and the remaining 1117 clones were made into glycerol stocks by adding filter-sterilized glycerol to a final concentration of 15% to each overnight culture. A portion of each glycerol stock was sent to the BC Cancer Agency in Vancouver, BC for unidirectional sequencing (M13 Forward primer), and the remainder was stored at −80°C for future potential functional analysis studies. As previously described [8], the *Ps*-infected DF library was sent to the BC Cancer Agency where 3517 clones were sequenced. The first 1213 sequences were unidirectional (M13 Forward primer) and the rest were bi-directional (M13 Forward and Reverse primers).

cDNA sequences were edited using Sequencher version 4.8 (Gene Codes Corporation, Ann Arbor, MI) by trimming the vector sequence and ambiguous base pairs at the ends of the sequences under the default settings of the program. The few remaining sequences that were less than 100 bp were deleted. For each of the two libraries, the sequence sets were assembled into contigs using the automatic assembly function in Sequencher. For the *Ps*-DF library, overlapping sequences (from both 5′ and 3′ ends of the same transcript) were evaluated and aligned into full consensus sequence contigs, but were reported as a singleton when no other cDNAs were part of the contig. All the unisequences were compared to the protein database at NCBI using the BLASTx algorithm [52]. Similarities were classified as hits indicating significant homology when the expected E-value was equal to or less than 10^−5^.

To identify open reading frames, all the *Ps* library sequences and the fungal matches from the *Ps*-DF library were analyzed using the ORF Finder [53]. Protein sequences corresponding to the putative open reading frames were searched using the NCBI BLASTp algorithms [52] to corroborate the identity of each ORF by confirming that matches were the same as for the BLASTx search and the predicted start codon aligned with that of homologous proteins. The translated sequences of cDNAs with positively identified start codons were analyzed for signal peptidase sites using SignalP 3.0 [54]. When both algorithms employed by this program (SignalP-NN and SignalP-HMM) predicted the same signal peptide and the SignalP-HMM signal peptide probability was ≥ 0.98, the gene was considered to be a candidate member of the secretome of *Ps*. We corroborated the hypothesized extracellular destination of these proteins using WoLF PSORT [55] for fungal proteins. When no ORF was detected because the 5′ portion of the gene was missing, the top three BLASTx matches were analyzed using SignalP, and if all three matches predicted a signal peptide (average SignalP-HMM signal peptide probability ≥ 0.98), the *Ps* sequence in question was also hypothesized to be a candidate member of the secretome of *Ps*.

Each unisequence was assigned to a functional category following the Munich Information Center for Protein Sequences Functional Categories (MIPS FunCat) annotation scheme [56]. The assignment of ESTs to these categories was aided in large part by COGEME [57]: a tBLASTx search (translated nucleotide database search using translated nucleotide query) of the COGEME database with the *Ps* sequences produced ranked matches as well as their functional classification group(s), which used a scheme adapted from that of MIPS [56]. The percentage of genes in each functional category was calculated as the number of individual sequences in that group divided by the total number of annotated fungal ESTs from the library. Relatively short ESTs (<350 bp) with no significant matches, poly-A tails, and negligible ORFs (none or <50 aa) were assumed to comprise 3′ untranslated regions, and were deleted from the data set. Although the start codon of longer ORFs with no significant matches could not be verified by comparison with homologues, those containing predicted signal peptidase sites were assumed to begin with the correct start codon, and were considered potential genes of interest.

### RNA extraction and cDNA synthesis for qRT-PCR

In total, 48 RNA samples were extracted from control mycelia and *Ps*-infected DF seedling roots over 8 time points: 12, 18, 24, and 36 h, and 2, 3, 5, and 7 days post-infection. Three independent biological replicates were prepared in triplicate for each time point. We used PFC-583 for qRT-PCR experiments. Each individual extraction included 10 to 14 seedlings for ~ 100 mg of plant tissue from infected roots, or approximately 5 petri plates of mycelia for ~ 80 mg fungal tissue. The control mycelia was grown on agar without cellophane, and mycelia were scraped from the plates using a liquid-nitrogen cooled spatula which was gently pulled across the surface of each plate to collect mycelia while avoiding any contamination from the agar. The RNeasy Plant mini kit (Qiagen) supplemented with Plant Isolation Aid (Ambion, Austin, TX) was used to extract total RNA. The extracted RNA samples were precipitated with 50% (v/v) 2.5 M LiCl at -20°C overnight, cleaned with 70% EtOH, resuspended in RNAse-free water, and treated with TURBO DNase (Ambion), and the SUPERase RNase Inhibitor (Ambion) was added to the recovered RNA. Total RNA was quantified using the NanoDrop spectrophotometer.

First-strand cDNA was synthesized from 500 ng of total RNA using the Transcriptor First-Strand cDNA Synthesis Kit (Roche Applied Science, Mannheim, Germany) following the manufacturer’s instructions.

### qRT-PCR

To analyze the expression of each candidate virulence gene, primer pairs were designed for each gene using Roche’s LightCycler Probe Design Software 2.0 (version 1.0; Roche). qRT-PCR was conducted with the synthesized cDNA using the LightCycler 2.0 System (Roche) according to the manufacturer’s instructions. Two microliters of 10× diluted cDNA sample was used as the template for each reaction, and 500 nM of each primer was added to the reaction mix. PCR cycling parameters were 95°C for 10 min; followed by 45 cycles of 95°C for 10s, 60°C for 10s, and 72°C for 10s; one cycle of 95°C to generate a melting curve; and finally, 40°C for cooling. Fluorescent PCR products were generated from cDNA using the LightCycler FastStart DNA MasterPLUS SYBR Green I kit (Roche) and relative quantification was performed using the LightCycler 4.1 software (Roche). Water was used as a no template control, and pure DF cDNA was used to ensure products were amplified from *Ps* rather than DF cDNA.

We considered actin and ubiquitin-conjugating enzyme E2 (*uceE2*) as candidate internal control genes. The variance of expression of each candidate control over each time-point during the 7-day infection were tested using GeNorm [58] and Normfinder [59]. GeNorm analysis calculated *uceE2* to be more stable than actin with a lower stabilization factor (M) of 1.18 compared to 1.47 respectively. GeNorm suggests genes with M values below 1.5 can be considered to have sufficiently stable expression [58]. NormFinder further validates the use of *uceE2*, with a lower stability value (0.444) than actin (0.494). Numerous qPCR studies have found ubiquitin conjugating enzyme to be a suitable internal control gene in plants [60] as well as in the oomycete, *Phytopthora parasitica*, during different life cycles including plant pathogenesis [61].

To determine the amplification efficiencies for the genes of interest and the *uceE2* gene, a concentrated cDNA sample was serially diluted over a 4 to 5 log range to cover the threshold cycles (Ct) observed in the experimental samples. qRT-PCR was performed for these dilutions and the Ct values were recorded at threshold and baseline parameters standardized for each of the transcripts. The data were plotted against the log of starting template concentration. The slope curve of the Ct cycles is dependent upon amplification efficiency (*E*) calculated by the equation *E* = 10[−1/slope] [62]. The expression level (R) of each gene of interest in relation to *uceE2* in control and infected samples was calculated from the Ct values and PCR amplification efficiencies using the following equation [62]:


To analyze and compare expression profiles for our two treatments (control versus infected) at different time points, transcript levels for control samples at 12 h were considered to be the basal levels during expression analysis and the corresponding transcript levels were given the value of 1.00. Ct values for all control and infected transcripts were calculated based on this basal value. As for each gene of interest, three separate biological replicates were each amplified in triplicate for the internal reference gene.

We performed an initial screen of qRT-PCR gene expression analysis for 86 putative virulence genes from *Ps* (see Additional files 3 and 4), testing single PCR reactions for each time point with one or two biological replicates. Based on this screen we focussed on genes that showed significant up- or-down regulation during infection, and for these genes we repeated qRT-PCR experiments for each of three biological replicates in triplicate. The primer sequences for genes that showed significant up- or down-regulation are shown in Additional file 6.

### Availabililty of supporting data

The data sets supporting the results of this article are included within the article and its additional files.

## Authors’ information

Abul K M Ekramoddoullah worked at the Pacific Forestry Centre with the other authors, but he is now retired.

## Electronic supplementary material

Additional file 1: **Genes from a cDNA library constructed using** ***P. sulphurascens*** **induced by exposure to Douglas-fir seedling roots.** 713 unique sequences from the *Ps* library, grouped into functional categories. (XLSX 93 KB)

Additional file 2: **Genes from a cDNA library constructed using** ***P. sulphurascens*** **-infected Douglas-fir seedling roots.** 270 unique sequences from the Ps-DF library, grouped into functional categories. (XLSX 43 KB)

Additional file 3: ***P. sulphurascens*** **genes potentially involved in pathogenicity.** Seventy-nine *Ps* genes considered to be candidate virulence factors. (XLSX 35 KB)

Additional file 4: ***P. sulphurascens*** **genes encoding predicted secreted proteins.** Eighty-two *Ps* genes with putative secretion signals. (XLSX 29 KB)

Additional file 5: **Partial gene sequence of** ***P. sulphurascens*** **putative 1,4 benzoquinone reductase.** Putative 1,4 benzoquinone reductase gene sequence obtained separately from the cDNA libraries. (DOCX 11 KB)

Additional file 6: **qRT-PCR primers for** ***P. sulphurascens*** **genes regulated during infection of DF seedlings and for two internal reference genes.** Primers corresponding to 16 *P. sulphurascens* genes up- or down-regulated during infection of DF and two internal reference genes. (XLSX 14 KB)

## Abbreviations

*Ps*: *Phellinus sulphurascens*
DF: Douglas-fir
EST: Expressed sequence tag
BLAST: Basic local alignment search tool
aa: Amino acids
dpi: Days post inoculation
hpi: Hours post inoculation
qRT-PCR: Quantitative reverse transcriptase-polymerase chain reaction.
