# Supplementary material for: Gene expression profiling of candidate virulence factors in the laminated root rot pathogen Phellinus sulphurascens
Source: BMC Genomics. 2014 Jul 17;15(1):603. doi: 10.1186/1471-2164-15-603 (PMC4117978; doi:10.1186/1471-2164-15-603)
Supplement: Supplementary file 5 — Additional file 5: Partial gene sequence of P. sulphurascens putative 1,4 benzoquinone reductase. Putative 1,4 benzoquinone reductase gene sequence obtained separately from the cDNA libraries. (DOCX 11 KB) [file 12864_2014_6292_MOESM5_ESM.docx]

**Additional File 5.** Partial gene sequence of *P. sulphurascens* putative 1,4 benzoquinone reductase (5’ -3’)

TCTATGTACGGCCACATAGCAAAGATGGCGGAGTCGGTCAAGGCTGGTGTCGAGTCTGCCGGTGGCGCAGTGACCATCTACCAGATTCCGGAGACGCTCGCTCCTGAGGCTCTCGCAAAGTTGTACGCCGCCCCCAAGCCCGACTACCCCATTATCGAGCCGGAAGAGCTTGAGAACTTCGATGCGTTCCTCTTGGGTATCCCCACTCGTTA
